# Learning-based autonomous vascular guidewire navigation without human demonstration in the venous system of a porcine liver

**DOI:** 10.1007/s11548-022-02646-8

**Published:** 2022-05-23

**Authors:** Lennart Karstensen, Jacqueline Ritter, Johannes Hatzl, Torben Pätz, Jens Langejürgen, Christian Uhl, Franziska Mathis-Ullrich

**Affiliations:** 1 Fraunhofer IPA, Theodor-Kutzer-Ufer 1-3, 68167 Mannheim, Germany; 2 Department of Vascular and Endovascular Surgery, University Hospital Heidelberg, Im Neuenheimer Feld 420, 69120 Heidelberg, Germany; 3 Fraunhofer MEVIS, Max-von-Laue-Str. 2, 28359 Bremen, Germany; 4 Institute for Anthropomatics and Robotics, Karlsruhe Institute of Technology, Engler-Bunte-Ring 8, 76131 Karlsruhe, Germany

**Keywords:** Endovascular intervention, Guidewire navigation, Deep reinforcement learning, Autonomous, Learning from scratch

## Abstract

**Purpose:**

The navigation of endovascular guidewires is a dexterous task where physicians and patients can benefit from automation. Machine learning-based controllers are promising to help master this task. However, human-generated training data are scarce and resource-intensive to generate. We investigate if a neural network-based controller trained without human-generated data can learn human-like behaviors.

**Methods:**

We trained and evaluated a neural network-based controller via deep reinforcement learning in a finite element simulation to navigate the venous system of a porcine liver without human-generated data. The behavior is compared to manual expert navigation, and real-world transferability is evaluated.

**Results:**

The controller achieves a success rate of 100% in simulation. The controller applies a wiggling behavior, where the guidewire tip is continuously rotated alternately clockwise and counterclockwise like the human expert applies. In the ex vivo porcine liver, the success rate drops to 30%, because either the wrong branch is probed, or the guidewire becomes entangled.

**Conclusion:**

In this work, we prove that a learning-based controller is capable of learning human-like guidewire navigation behavior without human-generated data, therefore, mitigating the requirement to produce resource-intensive human-generated training data. Limitations are the restriction to one vessel geometry, the neglected safeness of navigation, and the reduced transferability to the real world.

**Supplementary Information:**

The online version contains supplementary material available at 10.1007/s11548-022-02646-8.

## Introduction

On a global scale, the main cause of death is cardiovascular diseases (31.5%), in particular, ischemic heart (14.8%) and cerebrovascular (11.7%) diseases [1]. Endovascular, catheter-based interventions are the gold standard for treatment of numerous cardiovascular diseases [2]. In these interventions, a combination of a guidewire and catheter is navigated from an insertion point to the region of interest. The devices are inserted into the artery, typically in the groin or at the wrist, and manually navigated by twisting and pushing under image guidance with intraoperative fluoroscopy. The angled tip of the guidewire allows probing the desired branch at bifurcations. Typically, a bifurcation is mastered by rotating the guidewire, such that the angled tip enters the targeted vessel, and the remainder of the guidewire follows. When the guidewire sits firmly in the targeted vessel, the catheter is pushed over it. Additionally, the guidewire tip geometry can be modified by altering the overlap of guidewire and catheter. When advancing the guidewire through a vessel certain movement pattern can ease the navigation process, e.g., constantly wiggling the guidewire clockwise and counterclockwise can prevent the guidewire from entangling if the vessel meanders or smaller side vessels are present. After a successful navigation, the subsequent treatment through the catheter may be performed.

Improvements in treatment quality and speed can have a high impact on patients’ lives. Automation of catheter navigation has the potential to reduce radiation exposure of physicians when they must no longer stand near the patient. Even though radiation exposure can be reduced with measures of best practice, further reduction of radiation exposure is a sensible goal for the health of physicians [3, 4]. Furthermore, rural areas have reduced access to endovascular treatments compared to urban areas. A reliable autonomous system takes over the basic surgery and dexterity tasks, and the physician can focus on diagnosis and decision making, thus further enabling telesurgery and mitigating the supply gap [5, 6].

Endovascular robotics already transitioned from research to the industry in the form of telemanipulated manipulators, e.g., with the discontinued Magellan System (Hansen Medical, USA) or the currently available Corpath System (Corindus, USA) [7]. Among others, the main advantages are reduced radiation for the physicians [8] or telemedicine without direct patient visibility [9]. The Corpath System already incorporates automation of some movement patterns utilized by physicians in manual operations, e.g., the “wiggle” pattern [10], but no fully automated navigation is possible.

Current efforts to automate catheter and guidewire navigation can be divided into three categories: magnetically actuated, active, and passive catheters.

Control of a magnetic catheter tip in a closed-loop approach for eye surgery has been presented in [11]. The same principle is adapted to endovascular catheters in [12]. A recent development regarding autonomous magnetically actuated catheter navigation is the Advanced Robotics for Magnetic Manipulation System [13] that successfully positioned a magnetic catheter in a gelatinous phantom of a human torso with an embedded aorta using ultrasound images as feedback.

Fagogenis et al*.* [14] demonstrate autonomous navigation of a catheter with an active tip inside an in-vivo beating heart. Research regarding steerable catheters improves the feasibility and use cases of these devices, e.g., Gopesh et al*.* [15] fabricated and animal tested a steerable microcatheter for the endovascular treatment of cerebral disorders.

Passive catheters and guidewires are used much more frequently than the active or magnetic ones because there are a larger number of variants, they are less expensive and can be significantly smaller due to their simpler design. Furthermore, utilizing commercially available passive catheters enables faster clinical applicability as the invasive devices are already certified. Rafii-Tari et al*.* [16] propose an approach to learn optimum motion trajectories from expert demonstration and present their system in a phantom using electromagnetic tracking as feedback. Tercero et al*.* [17] demonstrate a system that navigates a catheter by following a pre-computed software map of movement commands. This software map may be adapted to a patient based on pre-interventional images.

Artificial intelligence embedded into robotics exhibits a high potential to improve the quality of surgical procedures. Diseases may be treated either through surgeons cooperating with intelligent robots or, in the future, by fully or semi-autonomous robotic systems [6, 18]. Therefore, approaches for automated navigation of passive guidewires or catheters emerge, which utilize deep learning. Chi et al*.* [19] investigate learning catheterization of the brachycephalic artery using expert demonstration with a generative adversarial imitation learning agent. Subsequently, a proximal policy optimization agent learns to catheterize the left common carotid artery. Both agents are trained in a type-1 aortic arch and evaluated in the type-1 and a type-2 aortic arch phantom using electromagnetic tracking as feedback. Zhao et al*.* [20] propose a convolutional neural network to estimate suitable manipulation actions. Additionally, an operating force mode estimator is trained to ensure safe operation. The estimated most suitable action is only executed if the operating force is normal. If the force becomes abnormal, predefined avoiding actions are executed. The neural networks are trained using demonstration data from expert surgeons. The controller is evaluated in a phantom with a gray-scale camera simulating X-ray imaging.

The combination of endovascular robotics and artificial intelligence has the potential to greatly advance autonomous navigation of endovascular catheters. The aforementioned approaches utilize human-generated data. However, availability of large-scale, high-quality, and correctly labeled data is limited and resource-intensive to generate. A controller trained from scratch without the necessity of data created by humans would circumvent the need for resource-intense data generation.

This work investigates the training of a neural network-based controller for endovascular navigation without human-generated data. The controller design builds upon prior work [21]. We investigate whether the controller can learn behaviors comparable to manual navigation. For this purpose, the controller learns the autonomous navigation of a guidewire from the insertion point to arbitrary targets within a vessel system. Exemplary the venous system of a porcine liver is utilized, as the porcine and human vessel system is similar, and pigs are often used for endovascular animal trials. The controller is trained and evaluated in a finite element simulation of one ex-vivo porcine liver specimen. For qualitative comparison, the same navigation is performed by a medical expert in a phantom modeled after the same specimen. The resulting successfully trained controller shows that the wiggle movement pattern has been learned as an optimal behavior without previous human demonstration. The main scope of this work is the investigation of the learned behavior patterns and maneuvers of the controller. The transferability of the simulation-trained controller to the real world is evaluated by performing autonomous navigation in the same ex vivo porcine liver specimen.

## Methods and materials

### Endovascular navigation task

**Fig. 1:**
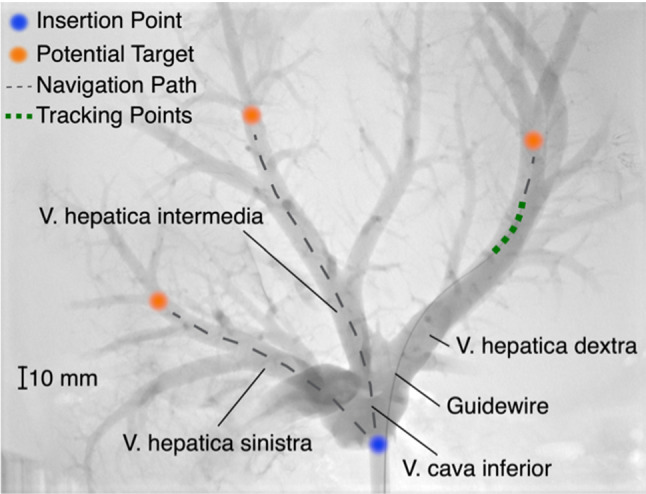
X-Ray of an ex-vivo porcine liver with contrast agent, a guidewire inserted in the vena hepatica dextra, possible target points including their navigation paths and the tracking points used as feedback

This work aims to autonomously navigate an endovascular guidewire through the veins of a porcine liver. The same specimen is utilized for in silico training and evaluation, manual phantom navigation, and ex vivo transferability testing. The guidewire is inserted in the vena cava inferior and enters the liver coming from the direction of the heart. The anatomy and nomenclature of the liver veins, the insertion point and possible targets with their navigation paths are illustrated in Fig. 1. The vena cava inferior separates into three main liver veins, i.e., vena hepatica dextra, vena hepatica intermedia, and vena hepatica sinistra. All three main hepatic veins bifurcate in several large and small side branches depending on the individual’s specific geometry. Here, we consider all larger branches, which are reachable by manual navigation. Smaller side branches are neglected due to mechanical limitations of the guidewire dexterity.

The guidewire (Terumo Radifocus Guide Wire M, angled, Stiff Type, 0.035″ Diameter, 3 cm flexible tip) is manipulated at its base outside the liver. During the navigation procedure, the guidewire position is used as feedback in the form of discrete tracking points, as illustrated in Fig. 1.

A navigation task starts at the vena cava inferior shortly before the trifurcation to the hepatic veins. The navigation target is a pre-selected point on the centerline of any branch. A navigation task is considered successful if the guidewire tip reaches the selected target within 40 s. In a manual preliminary experiment, 20 s was determined to be sufficient time for a navigation task which was doubled to allow exploration during training. The target is considered reached if the distance between the guidewire tip and the target is less than 15 mm.

The control task is characterized by a partially observable Markov decision process [22]. The Markov property requires that a single input state can describe the complete state of the given problem. However, the guidewire control is only partially observable as the two-dimensional X-ray images used as feedback during navigation provide the position of the guidewire in the image plane. The depth is only estimated from knowledge of the vessel anatomy. Information about the absolute rotation of the guidewire tip is not perceivable from the image as two images of the guidewire tip rotated at a specific angle upwards or downwards are ambiguous.

### Controller architecture and training procedure

**Fig. 2:**
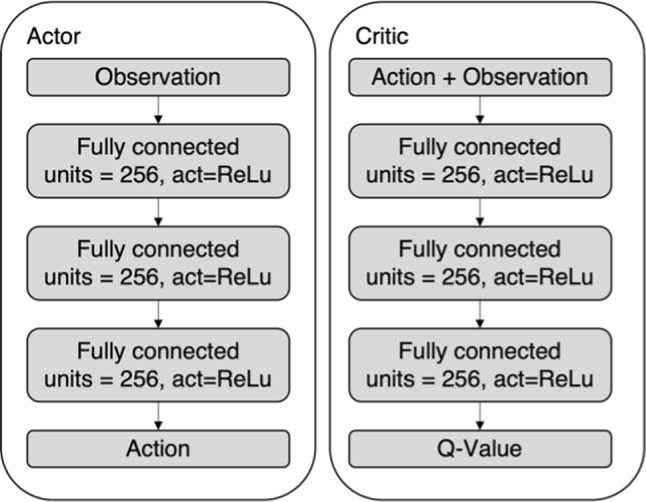
The structure of the actor and critic neural networks of the controller

The controller is a neural network, trained using Deep Deterministic Policy Gradients [23] with Hindsight Experience Replay [24]. Actor and a critic both have three fully connected layers of 256 Neurons, as illustrated in Fig. 2. The observation is defined as the current and the last two guidewire positions, the target position and the last two actions leading to the current state, thus creating a memory to make the rotation of the guidewire tip observable. By observing the movement direction of the tip, it can be estimated if it is turned upwards or downwards. The guidewire position is given as the tracking coordinates of five points on the guidewire described as (*x*, *z*)_*i*_ *i* = {1, 2, 3, 4, 5} relative to the insertion point where all points are spaced evenly and 1 mm apart and (*x*, *z*)_1_ is coincident with the guidewire tip. The target position is given as the target coordinates (*x*, *z*) of the current target. The action is given as the guidewire rotation and translation speed.

For all coordinate-based inputs, only the *x* and *z* coordinates are considered, representing the image plane. They are normalized between − 1 and 1 with (0, 0) indicating the geometric center of the porcine liver and − 1 and 1 being the lowest and largest coordinate on the liver, respectively. The action output of the actor network is the guidewire rotation and translation speed. Rotation and translation speeds are continuous values between − 6.28 rad/s to 6.28 rad/s and − 10 mm/s to 10 mm/s, respectively.

Hindsight Experience Replay provides best results with sparse rewards, reducing the required task-specific knowledge to set up the reinforcement learning environment, i.e., it is only necessary to detect whether the target is reached. Thus, the agent receives − 1 reward for every step and an additional + 1 reward for reaching the target. One step is defined as applying an action to the environment, receiving the observation, and subsequently calculating the next action. The episode is considered finished if the target is reached or 40 s have passed. One episode is defined as performing one navigation task. The control frequency is 7.5 Hz. For training and evaluation, targets are selected randomly on the branch centerlines and an episode always starts at the insertion point. The necessary insertion depth, depending on the distance between insertion point and target, has no influence on the maximum duration.

Every 750 training episodes, the performance is measured for 150 consecutive evaluation episodes. Performance is defined as the percentage of evaluation episodes where the controller successfully reaches the target. During training episodes, stochastic noise is added to the action calculation for exploration, but not during evaluation episodes. Training and evaluation are performed with 15 digital agents in parallel. Each agent periodically performs 50 training and 10 evaluation episodes.

### Training and evaluation environment

Training of the controller is solely performed in the simulation environment. The controller is evaluated in the same simulation environment. To examine real world transferability, the controller is additionally tested on a testbench in the ex vivo porcine liver used as basis for the simulation.

**Fig. 3:**
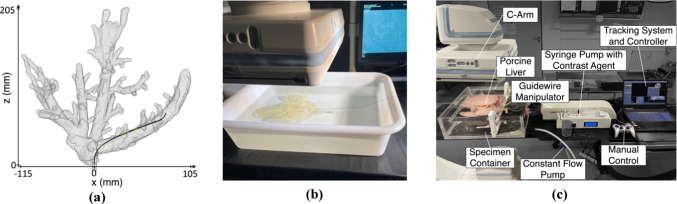
**a** Simulation model, **b** Phantom testbench and **c** Ex-vivo testbench for guidewire navigation

The simulation is modeled within the SOFA framework [25] using the BeamAdapter plugin [26]. Phantom walls are assumed rigid and the lumen empty. Friction between wall and guidewire as well as guidewire stiffness has been iteratively tuned to mimic guidewire behavior in the testbench. The simulation receives guidewire rotation and translation speed as input and the output is the guidewire position as coordinate points evenly spaced at 1 mm starting from the guidewire tip. The tracking position is received with a delay, which can be described by a normal distribution with a mean delay of 0.13 s and variance of 0.01 s. This mimics the processing delay of the testbench tracking system. A rendering of the simulation model can be seen in Fig. 3a.

To transfer the geometry of the porcine liver into the simulation environment, a CT scan (Artis Zeego, Siemens Healthineers) with contrast agent (potassium iodide) is performed. The veins are segmented in the CT image semi-autonomously [27]. The resulting segmentation is smoothed and transformed into a surface mesh.

The phantom testbench can be seen in Fig. 3b. The phantom consists of a rigid structure with lumens in the shape of the extracted vessel geometry, manufactured by stereolithography. A medical expert manually navigates the guidewire using continuous fluoroscopy (Artis Pheno, Siemens Healthineers) as feedback for comparison to the autonomous controller.

The ex vivo testbench is presented in Fig. 3c. The porcine liver is obtained from a local abattoir and prepared for guidewire navigation. A custom tube connector attached to the vena cava inferior allows guidewire insertion and the attachment of the constant flow pump. All other openings are closed by stitching or gluing.

The liver is placed inside a water-filled specimen container. A constant flow pumps water into the vena cava inferior to ensure that the veins are inflated. A customized guidewire manipulator translates and rotates the guidewire at its base. It allows for continuous rotation and a translation of up to 300 mm. The guidewire manipulator receives control commands by the trained controllers.

During the navigation task, continuous fluoroscopy (Artis Zeego, Siemens Healthineers) with a framerate of 7.5 images per second allows tracking of the guidewire. A tracking system evaluates each fluoroscopy image to extract the guidewire position. A recurrent convolutional neural network is utilized to segment the guidewire from the real-time fluoroscopy images, and a backprojection reconstructs the 3D shape of the guidewire. The output of the tracking system is the guidewire position as coordinate points evenly spaced and 1 mm apart from each other starting from the guidewire tip with a tracking error of approximately 1.3 mm.

## Results

**Fig. 4:**
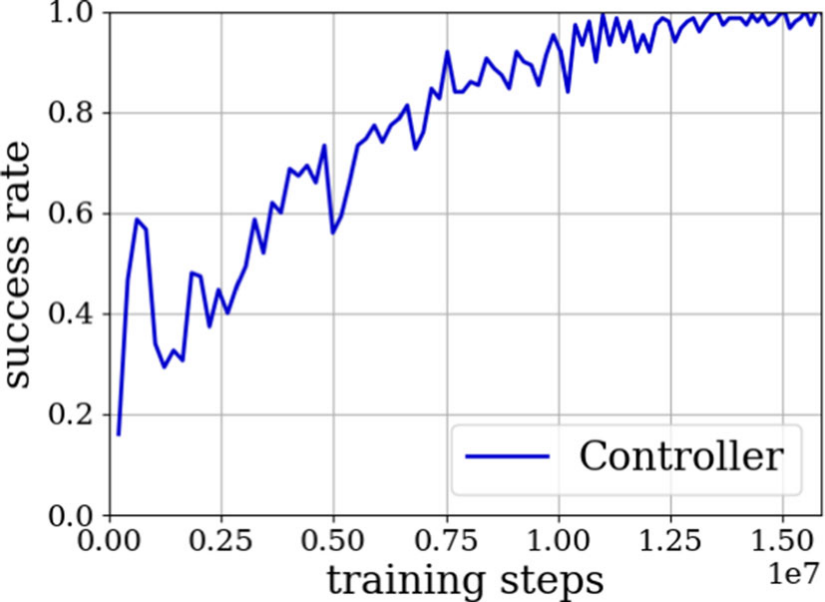
Success rate during the simulation training of the controller

Evaluation results during training of the controller are graphed in Fig. 4. As shown in the figure, the controller steadily improves. A success rate of 100%, meaning 150 out of 150 evaluation episodes with randomly sampled targets have been successful, is reached for the first time after 13.5 × 10^6^ training steps but continues to fluctuate between 90 and 100% for further evaluation steps.

**Fig. 5:**
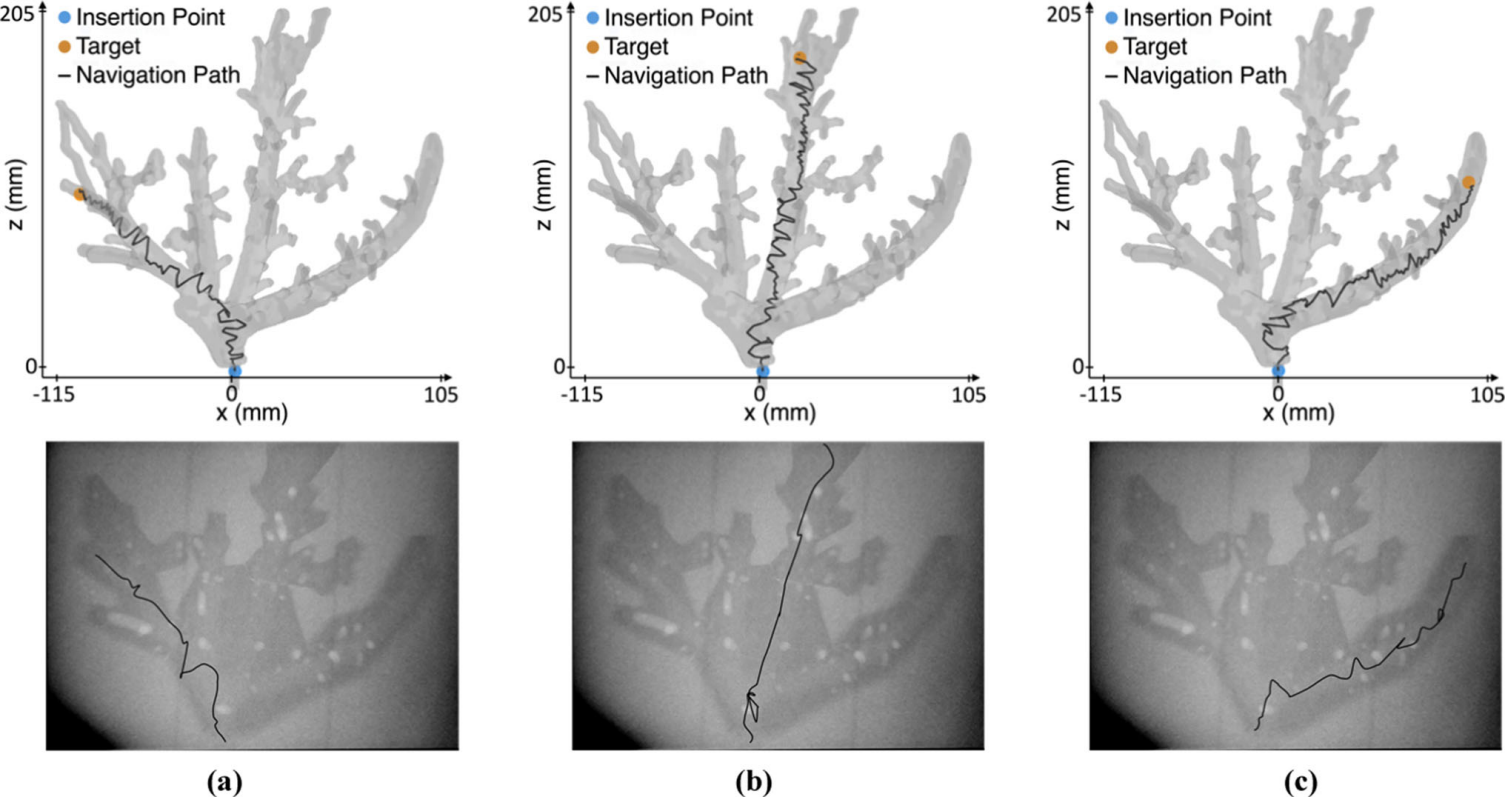
Trajectories of the guidewire tip for the navigation in the vena hepatica **a** sinistra, **b** intermedia and **c** dextra. Autonomous in-silico navigation is shown in the top row and manual phantom navigation in the bottom row

Figure 5 and Online Resource 1 illustrate the trajectory of the guidewire tip from the insertion point to the target by autonomous controller in silico and by a medical expert in the phantom. The autonomous controller maneuvers directly to the target, while the guidewire is continuously rotated alternately clockwise and counterclockwise, resulting in a wiggling behavior of the guidewire. In comparison, the medical expert utilizes the wiggle motion only where necessary, e.g., navigation in the vena hepatica intermedia shows very little rotation, while navigation in the vena hepatica dextra utilizes the wiggle motion frequently. Duration of the navigation procedure is similar. For the vena hepatica sinistra, intermedia and dextra, the autonomous and the manual navigation takes 14 s and 12 s, 17 s and 16 s, and 18 s and 17 s, respectively.

The controller, solely trained in the simulation, was evaluated in the ex vivo porcine liver specimen used to extract the vessel geometry for the simulation and phantom. 10 targets are specified in each of the three main liver veins: left (*sinistra*), center (*intermedia*) and right (*dextra*). The resulting number of successful navigation (*success*), failure due to probing the wrong branch (*wrong branch*) and failure due to entanglement of the guidewire tip while advancing the guidewire in the correct branch (*entanglement*) are presented in Table 1. The controller achieves a success rate of 30%, the failure rate of probing the wrong branch is 33.3%, and the failure rate of entanglement is 36.6%. Success rate and failure types vary between the branches.

**Table 1:** Evaluation results of the controller on the testbench stating the amount of successful navigation attempts and failures due to wrong branch navigation and entanglement

Branch	Sinistra	Intermedia	Dextra	Sum
Success	1	4	4	9
Wrong branch	6	0	4	10
Entanglement	3	6	2	11

## Discussion and conclusion

The navigation of guidewires in endovascular interventions is a dexterous task, and physicians and patients can benefit from automation. Machine learning-based controllers are promising to help master this task. However, training data provided by human operators are scarce and resource-intensive to generate. As it reduces the need for high-quality human-labeled data, learning with self-generated data is desirable. In this work, we have shown that a learning-based controller is capable of learning human-like guidewire navigation behavior without the usage of human-generated data. Even if no or limited data are available, a learning-based controller can perform high-quality guidewire navigation. Therefore, mitigating the requirement to use human-generated training data when training a reinforcement learning-based controller.

We have trained a neural network-based controller to navigate the venous system of a porcine liver from scratch without human demonstration. The controller was trained and evaluated in a simulation environment, compared to manual expert navigation in a phantom, and transferability to real-world applications was tested by applying the controller to the ex vivo porcine liver, which was the model for the simulation.

Concluding from the simulation results one may deduce that the controller design is capable of learning to navigate an endovascular guidewire through liver veins for a static setup. The controller manages to navigate to the target in 100% of the attempts. The reason for the fluctuation of success rate at the end of training is presumably due to the controller being updated further leading to worsening of the performance. The applied high learning rate allows fast learning at the beginning but can lead to suboptimal updates when the resulting neural network controller is close to its optimum. A solution may be utilizing an adjustable learning rate, which decreases during training. The controller proves to have learned the behavior of wiggling the guidewire alternately clockwise and counterclockwise, like the maneuver a physician applies in this situation. Interestingly, the maneuver was learned by the controller without being provided training data recorded from a physician that would indicate or teach this motion pattern. This shows that learning-based controllers are capable of learning motion patterns, which are inherently successful.

When transferred to the testbench with an ex vivo liver, the success rate of the controller drops to 30% of 30 given navigation tasks. Navigation failures by probing the wrong branch are most likely caused by registration inaccuracies, as registration for soft, deformable tissue without any visible landmarks remains a difficult task. The controller is not able to detect the failure from the guidewire position feedback. From the entanglement failures, it can be deduced that the controller does not learn general detection of entanglements and subsequent reaction to them.

There are limitations if one wants to transfer the results into clinical practice. In this study, only one vessel geometry was used, and the controller learns the movements to navigate this specific geometry instead of generalized guidewire navigation. In real-world scenarios, each patient has a unique vessel geometry and training a specific controller for each patient is impractical. A controller with a neural network structure and observations as presented is presumably not able to adapt to general geometries. Instead, either the geometry can be given as observation or neural network elements that can recognize the geometry during navigation need to be added, e.g., recurrent layers. We currently investigate generalization across different patient geometries with promising initial results. Additionally, the safeness of the guidewire navigation regarding wall contacts and applied forces is not considered in this study. Instead, only the navigation behavior to robustly reach the target is evaluated, as the sparse reward of the training process only values reaching the target. For a clinical transition, an analysis if the behavior is safe for the patient is necessary. Applicability of this simulation-trained controller in the real world is limited. Inaccuracies of the guidewire stiffness, the segmented vessel geometry, the interaction between guidewire and vessel walls, and shape variations of the ex vivo vessels, which are not considered in the simulation, lead to a significantly reduced real-world performance. By incorporating variations of the dynamics and observations, a controller may be trained more generally and should increase its real-world performance.

In view of future research fields, the reduced real-world performance indicates the importance of evaluation in the real world when developing endovascular robots and stresses the simulation-to-real gap. The utilization of phantoms can be an intermediate step to ease the transition. Furthermore, the guidewire becoming entangled represents a common problem, which should be a matter of future investigation. The controller should be able to detect entanglements and react appropriately. It could be beneficial to use the fluoroscopy image as controller input instead of guidewire tracking points to detect entanglements. Another area of research should be navigating a two-instrument system, by including a catheter. With the catheter, the shape and stiffness of the guidewire tip can be modulated, like a concentric tube robot. This additional degree of freedom may improve the navigation capabilities of the controller.

## Supplementary Information

Below is the link to the electronic supplementary material.

Supplementary file1 (MP4 140910 kb)
